# Infection prevention in personal services settings: Evidence, gaps and the way forward

**DOI:** 10.14745/ccdr.v45i01a01

**Published:** 2019-01-03

**Authors:** A Popalyar, J Stafford, T Ogunremi, K Dunn

**Affiliations:** 1 Centre for Communicable Diseases and Infection Control, Public Health Agency of Canada, Ottawa, ON; 2 Infection Prevention and Control Acute Care, Department of Health, Government of New Brunswick, Fredericton, NB

**Keywords:** personal services, infection, prevention, risk mitigation

## Abstract

**Background:**

Personal services is a continuously evolving industry that encompasses a variety of aesthetic treatments and personal enhancement services. Personal services are an important public health concern because delivery of service may pose potential health risks for both clients and workers. To date, there is a lack of evidence on the specific infection risks involved with personal services and the magnitude of these risks. While guidance and regulation of personal services settings do exist, they appear in varying degrees and complexity across Canada.

**Objectives:**

To summarize relevant literature on the risk of infections related to personal services; conduct an environmental scan of current provincial and territorial guidance and regulations; identify key risk mitigation measures; and summarize gaps and challenges.

**Methods:**

A working group of national experts in the field was established for consultation on key issues. A narrative literature review was conducted to summarize findings from relevant articles. Key questions and a literature search strategy were developed and articles were screened and critically appraised for eligibility. An environmental scan of key guidelines was also conducted to identify relevant legislation and guidance. Findings from both the narrative review and environmental scan were summarized to inform guidance and identify gaps.

**Findings:**

The review of the literature identified factors associated with increased risk of infection including inadequate training of personal services workers and non-compliance with established infection prevention principles. The environmental scan demonstrated that some guidelines have been developed by provincial/territorial ministries of health utilizing basic, generally accepted infection prevention principles. The established body of evidence that informs infection prevention and control recommendations is valid for health care settings; however there are factors to consider in extracting and applying such guidance to personal services settings. Major gaps and challenges remain in supporting both the advancement of infection prevention guidance and the development of enhanced regulatory frameworks, applicable to personal services settings in Canada.

**Conclusion:**

This review involved a comprehensive examination of relevant literature and provides a summary of issues addressing the risk of infection in personal services settings. There is a paucity of high quality evidence to support guidance, and findings reveal the need for further investigation and enhanced awareness of public health risks associated with personal services. Nonetheless, these findings can inform future research and the development of infection prevention and control guidelines and recommendations for such settings.

## Introduction

Personal services is a continuously evolving industry that encompasses a variety of aesthetic treatments and personal enhancement services, from non-invasive (such as hair and nail services) to invasive procedures such as microneedling and other body modification procedures. Many of these services intentionally or accidentally penetrate the body’s defences, posing an infection risk to clients and personal services workers.

There is little information on the infection risks specifically associated with these services. In addition, there is no national surveillance system related to complications of the personal services industry in Canada. While guidance and regulation of personal services settings do exist, the degree and complexity varies across Canada. With a lack of evidence related to disease acquisition in personal services settings, general principles of infection prevention are applied; these may not be directly applicable to the industry.

The objectives of this article are to summarize the available relevant literature on the risk of infections related to personal services; conduct an environmental scan of current provincial/territorial ministry of health guidance and regulations; outline generally accepted infection prevention principles relevant to personal services settings; and summarize major gaps and challenges. This article is intended to bring greater awareness from a public health perspective and be a resource for those considering the development of guidelines or regulations in this area.

A review of practice guidelines, recommendations, position papers, etcetera, produced by personal services and/or public health professional associations or by educational programs is beyond the scope of this article.

## Methods

### Expert Working Group

In 2013, an expert working group was established to inform the Public Health Agency of Canada (PHAC) of issues associated with personal services and to provide infection prevention guidance for this setting. Expertise from the field included public health nurses and inspectors and infection prevention and control professionals from Nova Scotia, New Brunswick, Ontario, Manitoba and Alberta. The expert working group reviewed findings from the literature search and environmental scan.

### Literature review

A narrative literature review was conducted to determine and summarize findings from relevant studies on the risk of infections related to personal services and inform the development of guidance. Key questions addressed prevalence, infection risk factors and infection prevention strategies for the three categories of personal services: piercing; other invasive services; and non-invasive services. The Health Library (Health Canada) undertook a comprehensive literature search using PubMed, Embase, Global Health, Ovid MEDLINE, Ovid MEDLINE Daily and Ovid OLDMEDLINE databases for studies published from January 1999 to December 2016.

The search was limited to studies in English and French with no filters applied, which would limit retrieval by study design. The full texts of all retrieved studies were manually screened to identify studies that reported on the receipt of one or more of the following categories of services:

- Body modification (i.e., ear/body piercing, body/eyeball tattooing, micropigmentation, scarification, tongue splitting, beading, jewellery implants, ocular jewellery, branding)
- Injections (i.e., fillers)
- Cosmetology (i.e., aesthetics, hair dressing/barber services, shaving, microdermal abrasion, facials, artificial nails, manicures, pedicures, make-up, face painting, waxing, electrolysis); and/or
- Other personal services (i.e., health spa/skin clinic, mud/steam bath, laser service including hair removal/skin resurfacing, massage, tanning, aromatherapy, teeth whitening, colonic irrigation, flotation tanks/water therapy)

**AND** the development of one or more of the following:

- Skin/soft tissue infection
- Bloodborne infection (e.g., hepatitis B, hepatitis C, HIV, other); and/or
- Systemic infection (e.g., endocarditis, septicemia, other)

### Environmental scan

An environmental scan of ministry of health websites was conducted to identify provincial and territorial guidelines, standards and regulations to do with personal services.

Guiding principles for infection prevention and control, as applicable to personal service settings, were identified and summarized.

## Summary of findings

### Expert Working Group

Challenges and gaps identified by public health inspectors and infection prevention and control professionals highlighted the need for increased awareness as well as improved guidance and regulations.

### Literature review

Of the 729 papers identified for preliminary screening, 555 were reviews or abstracts and were therefore excluded. A further 92 papers did not meet the search criteria outlined in the scope. A critical appraisal of the remaining 82 studies was accomplished using the PHAC *Infection Prevention and Control Guidelines Critical Appraisal Tool Kit* (1), and a further 31 papers were eliminated due to flaws in methodology (n=16) or analysis of results (n=15). This resulted in a total of 51 papers on the risk of infections related to personal services.

### Risk of infection and transmission

The risks identified in the literature were quite varied. Information relevant to infection risks and the magnitude of these risks specific to Canadian personal services settings were limited however, a number of studies identified factors associated with increased risk of infection in personal services settings in other countries:

- Inadequate training and skill level of personal services workers (resulting in poor infection prevention control practices) (2,3)
- Poor or non-compliance with generally established infection prevention practices (resulting in individual cases or wider outbreaks of infection) (4–7)

Specific findings related to breaches or non-compliance with recommended infection practices include:

- Improper glove use (8)
- Improper cleaning of the environment (9,10)
- Improper cleaning, disinfection and sterilization of tools or equipment (2,7,8,10–20)
- Use of non-sterile instruments for invasive procedures (8,17–20)
- Use of contaminated water, ink, supplies or equipment (6,7,21–32)
- Pre-existing health status of the clients (33–39)
- Failure to provide adequate after care instructions (40,41)
- Deficiencies in the physical layout and inadequate infection prevention and control practices, including lack of hand washing facilities and/ or with no potable water (8)

Studies showed that infections associated with personal services may be bacterial (38,42–46), viral (47–52) or fungal (53). The risk for transmission of bloodborne viruses within personal services settings is impacted by knowledge of and/or adherence to effective, established infection prevention practices (54–58). Specific risk factors associated with exposure to bloodborne infections during personal services procedures include:

- Potential contact with blood when sharps containers are not placed within reach, leading to unnecessary handling of contaminated sharps and injuries; improper disposal of sharps, by, for example, repackaging used sharps or discarding them in the regular garbage
- Cross-contamination of instruments and surfaces
- Re-use of disposable instruments and equipment such as razors and styptic pencils
- Inadequate disinfection and sterilization of equipment
- Inadequate management of cuts and abrasions on personal services workers
- Inconsistent hand hygiene and glove use
- Lack of knowledge about appropriate procedures and routes of transmission of bloodborne pathogens
- Lack of vaccine-induced protection (e.g., for hepatitis B)

### Environmental scan

The environmental scan was limited to provincial/territorial ministry of health websites to identify relevant legislation, regulations and approved guidelines, practices and standards. Guidance and regulations for personal services settings exist in varying degrees and complexity across Canada (**Table 1**). A review of practice guidelines, recommendations, position papers, etc. produced by personal services and/or public health professional associations or by educational programs was beyond the scope of the scan.

**Table 1:** Summary of published provincial and territorial personal services guidelines, standards, protocols, acts and regulations

Province/territory	Guidelines, standards, protocols and/or other	Acts, regulations and/or bylaws
Newfoundland and Labrador	N/A	*Personal Services Act*, 2012 (59)
Prince Edward Island	Guidelines for Tanning Salon Owners and Operators, 2011 (60)^a^	*PEI Public Health Act*, 2018 (61)
Nova Scotia	Salon and Spa Compliance Handbook, no date (62)	*Safe Body Art Act*, 2011 (63) *Health Protection Act*, 2016 (64) *Safe Body Art Regulations*, 2018 (65)
New Brunswick	N/A	*New Brunswick Bill 56 Public Health Act*, 1998 (66)
Quebec	Tattooers and Piercers: Protect Your Client and Yourself Against HIV and Hepatitis B and C, 1999 (67)	N/A
Ontario	Infection Prevention and Control Best Practices for Personal Services Settings, 2009 (68) Infection Prevention and Control Disclosure Protocol, 2018 (69) Infection Prevention and Control Complaint Protocol, 2018 (70) Personal Service Settings Guideline, 2018 (71) The Ontario Public Health Standards: Requirements for Programs, Services, and Accountability, 2018 (72)	*Health Promotion and Protection and Promotion Act, Ontario Regulation 136/18: Personal Service Settings*, 2018 (73)
Manitoba	Personal Services Facility Guideline, 2013 (74)	N/A
Saskatchewan	Personal Service Facility Best Management Practices, 2014 (75)	*The Health Hazard Regulations*, 2002 (76)
Alberta	Health Standards and Guidelines for Tattooing, 2002 (77) Health Standards and Guidelines for Body and Ear Piercing, 2002 (78) Health Standards and Guidelines for Barbering and Hairstyling, 2002 (79) Health Standards and Guidelines for Esthetics, 2002 (80) Health Standards and Guidelines for Electrolysis, 2002 (81)	*Public Health Personal Services Regulation*, 2003 (82)
British Columbia	Guidelines for Personal Services Establishments, 2017 (83) Guidelines For Body Modification, 2017 (84) Guideline for Personal Services Offered at Tradeshows, 2016 (85) Guidelines for Floatation Tanks, 2016 (86) Laser Hair Removal Devices: Safety Guidelines for Owners/Operators, 2005 (87) Microblading Services in Personal Service Establishments – Fact Sheet for Operators, 2017 (88)	*Public Health Act, Regulated Activities Regulation*, 2011 (89)
Yukon	Personal Service Premises Inspection Model, 2013 (90)	*Public Health Act – Personal Service Establishment Regulations*, 1984 (91)
Northwest Territories	Standards for Personal Service Establishments, 2012 (92)	*Public Health Act – Personal Services Establishment Regulations*, 2013 (93)
Nunavut	N/A	*Public Health Act*, 2016 (94) *Barber Shops and Beauty Salons Regulations*, 1990 (95)

Abbreviation: N/A, not applicable;

^a^ Limited focus on infection prevention control

### General risk mitigation measures

The scope of personal services is very broad and different services and settings may require different infection prevention guidance. Generally accepted key measures that minimize infection risk are summarized in **Table 2**. Consistent application of infection prevention practices and precautions help prevent the acquisition and transmission of infections. The general infection prevention principles outlined in Table 2 are not comprehensive and are based on core infection prevention principles as identified in the PHAC guideline: *Routine Practices and Additional Precautions for Preventing the Transmission of Infection in Healthcare Settings* (96).

**Table 2:** General infection prevention principles to mitigate risk of exposure to infections in personal services settings

Risk Mitigation Measure	Additional Context
Administrative controls	Provide an infrastructure of protocols and practices intended to prevent the risk of infection to personal services workers and clients in personal services settings Administrative controls include infection prevention policies and procedures; education and training (along with readily available resources such as instructions and manuals); proper use of equipment and instruments; monitoring compliance with infection prevention practices; appropriate occupational health and safety practices (e.g., worker immunization); and documentation and record keeping (e.g., records of disinfection and sterilization) in accordance with municipal and/or provincial/territorial standards and legislation
Risk assessment	Must be performed before undertaking any personal service activity to evaluate the risk of infection or cross-contamination associated with an activity and to implement appropriate prevention measures Includes determining the potential for contact with blood, body fluids and non-intact skin for the worker or client, exposure to mucous membranes and exposure to contaminated equipment or surfaces
Hand hygiene	Single most important measure for preventing the transmission of microorganisms in all settings Should be performed (as recommended in the PHAC *Hand Hygiene Practices in Healthcare Settings* guideline and local or provincial/territorial guidelines) using either an alcohol-based hand rub or liquid soap and water if hands are visibly soiled (97) Gloves are not a substitute for hand hygiene
Environmental cleaning and disinfection	Helps reduce the contamination of surfaces, decreasing the risk of transmission of microorganisms that may lead to infections in clients or workers Manufacturer’s directions for use and contact times for cleaning and disinfection products must be followed Low-risk surfaces (e.g., tables covered with a single-use towel, hairdressing chairs or sinks for hair washing) are less likely to contribute to an infection as they typically come into contact only with intact skin. These surfaces should be cleaned immediately when they become visibly soiled and at least once per day (98) Higher-risk surfaces (e.g., manicure/pedicure tables not covered with a single-use towel, counters used to prepare materials, equipment for procedures or foot baths) are more likely to be contaminated from contact with non-intact skin and blood and/or other body fluids. These surfaces should be cleaned and disinfected between clients and when surfaces are visibly soiled (98)
Single-use devices and products	Single-use devices and products should be used wherever possible and, where applicable, lot numbers and expiry dates should be checked prior to use Single-use devices and products must be discarded after one use: they must not be reprocessed, reused or kept in the personal services setting for future use with either the same client or a different client
Reprocessing reusable devices	Level of reprocessing required for a specific reusable device depends on the device’s intended use and the risk of infection to the client All reusable devices require meticulous cleaning prior to disinfection or sterilization Reusable devices used in the provision of services to clients must be reprocessed according to manufacturer instructions for cleaning, disinfection and/or sterilization and should adhere to the most current reprocessing standards from the Canadian Standards Association. In the absence of specific manufacturer’s instructions, decisions around reprocessing should be based on provincial/territorial best practice recommendations (96) or determined based on Spaulding’s classification (99)

Abbreviation: PHAC; Public Health Agency of Canada

### Gaps and challenges

Following the review of findings from the narrative review and environmental scan as well as discussions with the expert working group, a number of gaps and challenges were identified. These are summarized in **Table 3**.

**Table 3:** Gaps and challenges both related to infection prevention and outside the scope of infection prevention

Gap/Challenge related to:	Context
Related to infection prevention	
Setting	**Health care guidelines and standards for infection prevention are not directly applicable to personal services settings** Personal services settings serve a healthier client base compared to most health care settings Personal services settings are often small businesses; feasibility of implementing guidelines/standards is an important consideration The physical layout and design of these settings can contribute to infection prevention issues. Personal services are no longer only offered in traditional commercial settings; they now include mobile, home-based, mall kiosk and special-event settings. There are limited guidelines and standards in this industry to address these issues directly. Where guidelines and standards do exist, they are mostly developed from the perspective of permanent commercial settings (e.g., stores in retail spaces) and may not be applicable to alternate settings
	**Limited and poor quality literature and data on risk of infection and the burden of illness associated with personal services settings** No Canadian research published for infection prevention in personal services settings; data obtained from poor quality evidence such as case reports
	**Recommendations for cleaning and disinfection, including recommendations for products used to clean and disinfect, exist in varying degrees and complexities** Practices for cleaning and disinfection are inconsistent Availability and purchase of standardized disinfection products can be a challenge in community practice A similar challenge exists for antiseptic products. Some settings wish to use alternative products that may not be appropriate for antisepsis
Infection prevention education and training	**Education and training of workers on infection prevention is not feasible (or enforceable) in many personal services settings**
	**Workplace and practice audits by personnel trained in infection prevention are often not available to personal services settings**
Outside the scope of infection prevention	
Legal infrastructure	**A consistent definition of personal services across jurisdictions is difficult to achieve** as this is a continuously evolving industry. Lists of procedures that can be offered in these settings exist; however they are quickly outdated and are inconsistent across jurisdictions
	**Jurisdictional guidance and/or regulation regarding acceptable procedures and standards may be limited for non-regulated workers.** There are questions around the type of procedures acceptable for delivery by personal services workers versus delivery by health care professionals
	**Health care professionals are providing services in medical spa settings; this has created a grey area for public health inspectors.** While the practice of the personal services worker falls under the jurisdiction of the professional regulatory body, the service delivery setting itself can require public health inspection if located outside the mandate of a health authority
Client safety	**Chemicals and devices used in personal services settings can cause injuries such** as those associated with the application of energy (e.g., lasers, fat freezing, cryotherapy chambers, plasma pens) and injections (e.g., mesotherapy). Health care organizations have protocols, procedures, and oversight in place to ensure devices and products are used safely and to address any injuries; many personal services settings do not have this type of infrastructure
	Health Canada licences medical devices, products and chemicals that can be sold in Canada, but **other substances that may not be licensed for use in Canada are available for purchase internationally via the internet**
	**The public do not consistently have access to inspection reports** that would assist them in their choice of personal services setting
Worker skill and knowledge	**There is uncertainty regarding scope of practice**, in particular for workers without a professional regulatory body
	**Many workers do not have formal standard education and training in the services they provide.** They may be self-taught or learn from another worker
	**Most personal services settings require a license to operate, but not all workers have their practice regulated by a professional college/association**

## Discussion

There are concerns about infection prevention in the personal services industry. This article describes some of the concerns from a public health perspective, based on published studies and an environmental scan of guidelines and regulations available on provincial/territorial ministry of health websites. In addition, the gaps and challenges presented are a preliminary list of major issues as identified by the external expert working group, and do not encompass the full breadth or complexity of issues faced by public health in general.

There were notable limitations with the results of the literature review, in terms of comparability and applicability of available evidence to the Canadian context of personal services settings. This includes challenges with the quality of evidence, and limitations to extraction of data from case reports, self-reports, laboratory sampling, medical records and survey questionnaires. General principles and core elements for infection prevention are available from an established and recognized body of evidence that informs recommendations for practice in health care settings; however, there are challenges when applying measures from one setting to another. When extracting specific guidance for health care settings and adapting to personal services settings, some measures may not be relevant or directly apply.

This industry continues to evolve, with emergence of new procedures and services across a range of personal settings. The majority of publications and reports available focused on tattooing and piercing; however, a number of areas of personal services have no published information. Examples include body modification (tongue splitting, branding and scarification), nail salons and laser device uses for body enhancement. There is a need for further investigation to reflect the broad range of services and risks for exposure and transmission of infections in the Canadian context.

The feasibility of implementing infection prevention standards can be a challenge for alternate small business settings. The physical layout and design of these settings can contribute to infection prevention issues, there is limited evidence and data on the risk of infection in these settings, practices for cleaning and disinfection are inconsistent, and worker education and training on infection prevention are also limited depending on available resources.

In relation to the legal infrastructure, difficulties in defining personal services spill over into jurisdictional and regulatory issues and create grey areas in public health. Client safety is a major concern, particularly in the use of chemicals and devices in personal services settings. There is a need for standardized and consistent education and training of personal services workers.

While some organizations, such as the National Collaborating Centre on Environmental Health and the Canadian Institute of Public Health Inspectors, continue to examine and make efforts toward addressing issues related to personal services, further work is needed in this area. Canadian studies on infection prevention in personal services settings is recommended to provide information on the transmission pathways and risk of infections, and allow for assessment of burden of illness related to personal services settings in Canada. A continuously evolving industry also requires keeping an eye out for new services while working on legislation, regulation, guidelines, licensing and public education.

### Conclusion

Personal services is a continuously evolving industry that encompasses a variety of aesthetic treatments and personal enhancement services, including procedures that range from non-invasive to more invasive, with associated risk of infection to clients and workers. This overview includes a summary of current regulations and guidelines across provincial and territorial jurisdictions. Findings were informed by the contribution of experts in the field, in addition to results from the narrative review and environmental scan.

Despite limitations to evidence on the specific infection risks associated with these services, reports and publications do indicate contributing factors and findings that can be used to inform risk mitigation strategies. At the current time, there is no established surveillance system for data related to complications associated with the personal services industry in Canada. This summary identifies gaps and challenges to bring greater awareness from a public health perspective, and opportunities to address public health concerns through policy, regulation and guidelines, in an effort to promote and monitor best practices for the health of Canadians.
